# Colony-stimulating factor (CSF) 1 receptor blockade reduces inflammation in human and murine models of rheumatoid arthritis

**DOI:** 10.1186/s13075-016-0973-6

**Published:** 2016-03-31

**Authors:** Samuel Garcia, Linda M. Hartkamp, B Malvar-Fernandez, Inge E. van Es, Haishan Lin, Justin Wong, Li Long, James A. Zanghi, Andrew L. Rankin, Emma L. Masteller, Brian R. Wong, Timothy R. D. J. Radstake, Paul P. Tak, Kris A. Reedquist

**Affiliations:** Department of Experimental Immunology, Academic Medical Center, University of Amsterdam, Amsterdam, The Netherlands; Department of Clinical Immunology and Rheumatology, Academic Medical Center, University of Amsterdam, Amsterdam, The Netherlands; Laboratory of Translational Immunology and Department of Rheumatology and ClinicalImmunology, University Medical Center Utrecht, Utrecht, The Netherlands; Five Prime Therapeutics, Inc., Two Corporate Drive, South San Francisco, CA USA; Present address: GlaxoSmithKline, Stevenage, UK; Present address: Cambridge University, Cambridge, UK

## Abstract

**Background:**

CSF-1 or IL-34 stimulation of CSF1R promotes macrophage differentiation, activation and osteoclastogenesis, and pharmacological inhibition of CSF1R is beneficial in animal models of arthritis. The objective of this study was to determine the relative contributions of CSF-1 and IL-34 signaling to CSF1R in RA.

**Methods:**

CSF-1 and IL-34 were detected by immunohistochemical and digital image analysis in synovial tissue from 15 biological-naïve rheumatoid arthritis (RA) , 15 psoriatic arthritis (PsA) and 7 osteoarthritis (OA) patients . Gene expression in CSF-1- and IL-34-differentiated human macrophages was assessed by FACS analysis and quantitative PCR. RA synovial explants were incubated with CSF-1, IL-34, control antibody (Ab), or neutralizing/blocking Abs targeting CSF-1, IL-34, or CSF1R. The effect of a CSF1R-blocking Ab was examined in murine collagen-induced arthritis (CIA).

**Results:**

CSF-1 (also known as M-CSF) and IL-34 expression was similar in RA and PsA synovial tissue, but lower in controls (*P* < 0.05). CSF-1 expression was observed in the synovial sublining, and IL-34 in the sublining and the intimal lining layer. CSF-1 and IL-34 differentially regulated the expression of 17 of 336 inflammation-associated genes in macrophages, including chemokines, extra-cellular matrix components, and matrix metalloproteinases. Exogenous CSF-1 or IL-34, or their independent neutralization, had no effect on RA synovial explant IL-6 production. Anti-CSF1R Ab significantly reduced IL-6 and other inflammatory mediator production in RA synovial explants, and paw swelling and joint destruction in CIA.

**Conclusions:**

Simultaneous inhibition of CSF1R interactions with both CSF-1 and IL-34 suppresses inflammatory activation of RA synovial tissue and pathology in CIA, suggesting a novel therapeutic strategy for RA.

**Electronic supplementary material:**

The online version of this article (doi:10.1186/s13075-016-0973-6) contains supplementary material, which is available to authorized users.

## Background

Rheumatoid arthritis (RA) is a chronic autoimmune disease that affects the peripheral joints, leading to joint deformity and destruction [1]. Synovial macrophages, through their ability to release inflammatory cytokines, matrix metalloproteinases (MMPs), chemokines, reactive oxygen and nitrogen intermediates, and prostanoids, play a central role in the pathological process of RA [2]. Importantly, macrophage numbers and cytokine products are associated with disease activity and disease progression in RA, and decreases in synovial sublining CD68+ macrophages represent a sensitive biomarker of effective treatment [2]. Therefore, strategies specifically designed to target the activation, survival, or pro-inflammatory differentiation of macrophages in synovial tissue may be of therapeutic benefit in RA [2, 3].

Colony-stimulating factors (CSF) and functionally related cytokines regulate myeloid lineage cell development, proliferation, survival, mobilization, differentiation, and activation in both health and disease [4]. Granulocyte-macrophage CSF (GM-CSF) plays an important role in promoting the differentiation of granulocytes and macrophages from hematopoietic precursors [4]. During inflammation this property of GM-CSF may be important for the sustained recruitment of immature monocytes to affected tissues [5–7]. GM-CSF may also directly contribute to inflammation by polarizing macrophages into a pro-inflammatory phenotype, and participating in cytokine networks with other pro-inflammatory cytokines such as TNF and interleukin (IL)-1β [8, 9]. Administration of exogenous GM-CSF has been demonstrated to exacerbate collagen-induced arthritis (CIA) in mice, while mice deficient in GM-CSF expression are protected against disease in this model of arthritis [6, 10–13]. Additionally, neutralizing antibodies (Abs) against GM-CSF display both prophylactic and therapeutic efficacy in experimental arthritis [6, 14]. The direct relevance of these findings to RA is exemplified in recent clinical trials in which a blocking Ab directed at the alpha chain of the GM-CSF receptor has demonstrated safety and efficacy [15, 16].

Colony-stimulating factor-1 (CSF-1), through binding to the tyrosine kinase receptor CSF1R (also known as c-fms), also promotes the survival, proliferation and differentiation of myeloid cells, including monocytes, macrophages and osteoclasts [4, 17]. CSF-1 plays a distinctly different role from GM-CSF in myelopoiesis, acting as a survival and differentiation factor for mature circulating monocytes (Ly6C^lo^ in mice, CD16^+^ in humans) and resident tissue macrophages [6, 17, 18]. In RA, CSF-1 is produced mainly by synovial endothelial cells, but in vitro data indicate that synovial fibroblasts and chondrocytes stimulated with TNF or IL-1β also produce CSF-1 [19–21]. Animal models have shown that exogenous administration of CSF-1, like GM-CSF, exacerbates the incidence and severity of CIA, while anti-CSF-1 Ab, genetic deletion of CSF-1, and pharmacological inhibitors of CSF1R reduce the severity of experimental arthritis [11, 12, 22, 23]. CSF-1 also has an essential function in joint destruction, as CSF-1 is required for osteoclastogenesis and TNF-induced osteolysis [24, 25].

IL-34 was recently discovered as a novel ligand of CSF1R through its ability to support monocyte viability [26]. CSF-1 and IL-34 share structural, but not sequence homology, and have largely overlapping effects on CSF1R downstream signaling, regulation of monocyte survival, macrophage polarization, and osteoclastogenesis [27–32]. However, during murine development, IL-34 plays a non-redundant role in the generation of Langerhans cells and microglia [33, 34]. Recent studies have reported that IL-34 levels are elevated in the serum, synovial fluid, and synovial tissue of RA patients, and like CSF-1, IL-34 is induced by TNF and IL-1β in RA fibroblast-like synoviocytes (FLS) [35–38]. The goal of this study was to determine the relative contributions of CSF-1 and IL-34 to CSF1R-dependent inflammation in RA.

## Methods

### Patients and tissue donors

Synovial biopsies were obtained by needle arthroscopy as previously described from clinically active joints in patients with RA (n = 15), psoriatic arthritis (PsA) (n = 15), or osteoarthritis (OA) (n=7), as previously described [39]. RA and PsA patients fulfilled the 1987 American College of Rheumatology criteria for RA and the classification of psoriatic arthritis study (CASPAR) criteria, respectively [40, 41]. Patient clinical characteristics are detailed in Additional file 1: Tables S1 and S2. All patients gave their written informed consent prior to study inclusion, and this study was approved by the Medical Ethics Committee of the Academic Medical Center, University of Amsterdam, and in accordance with the Declaration of Helsinki.

### Immunohistochemical and digital imaging analysis

Serial sections from six different TissueTek-embedded biopsy samples per patient were cut with a cryostat (5 μm), fixed with acetone, and endogenous peroxidase activity blocked with 0.3 % hydrogen peroxide in 0.1 % sodium azide/phosphate-buffered saline. Sections were stained overnight at 4°C with Abs against CSF-1 (R&D), IL-34 (M4, provided by Five Prime Therapeutics Inc.) and CD68 (Dako). Equivalent concentrations of irrelevant mouse monoclonal Abs were used as negative controls. Sections were then washed and incubated with goat anti-mouse-horseradish peroxidase (HRP) (Dako), except in the case of anti-IL-34 Ab, which was followed by incubation with biotinylated tyramide and streptavidin-HRP. Sections were developed with amino-ethylcarbazole (AEC, Vector Laboratories), counterstained with Gill’s hematoxylin (Klinipath), and mounted in Kaiser’s glycerol gelatine (Merck). Stained sections were analyzed by computer-assisted image analysis using the Qwin analysis system (Leica) as previously described in detail [42]. Values of integrated optical densities (IOD)/mm^2^ were obtained and corrected for the total number of nuclei/mm^2^. Data were presented as the number of positive cells/mm^2^ for quantitative analysis of cell-type-specific markers.

### Monocyte purification and macrophage differentiation

Human mononuclear cells were isolated from volunteer donor blood buffy coats (Sanquin), peripheral blood from healthy controls and RA patients, and from synovial fluid of RA patients by gradient centrifugation with Lymphoprep (Axis-Shield PoPAS) and monocytes were further isolated by Percoll gradient separation (GE Healthcare). Monocytes were differentiated into macrophages in IMDM/10 % fetal calf serum (FCS) supplemented with 100 μg/ml gentamycin (Invitrogen), in the presence of human GM-CSF (5 ng/ml, R&D Systems), CSF-1 (25 ng/ml, R&D Systems), or IL-34 (25 ng/ml, provided by Five Prime Therapeutics Inc.) for 7 days.

### Flow cytometry

Macrophage purity and differentiation were assessed by flow cytometry (FACS Canto Flow Cytometer, BD Biosciences). Fluorochrome-labeled monoclonal Abs against CD14 (eBiosciences), CD206 and CD163 (both from BD Pharmingen), CD64 (Biolegend), and equivalent concentrations of isotype control Abs were used. Before staining, Fc receptors were blocked with 10 % human serum (Lonza). Data were analyzed with FlowJo Flow Cytometry Analysis software (Tree Star). Values were expressed as the ratio of the geometric mean fluorescence intensity (geomean) of the marker of interest over that of the isotype control.

### Macrophage cell death and viability

Macrophage cell death was assessed by Annexin V-propidium iodide (PI) staining. GM-CSF-, IL-34-, and CSF-1-differentiated macrophages in peripheral blood from healthy donors and in peripheral blood and synovial fluid from RA patients were stained with Annexin V (1:100 dilution, iQ products) and PI (1:100 dilution, iQ products) and measured using a FACS Canto Flow Cytometer. Data were analyzed with FlowJo Flow Cytometry Analysis software. Values were expressed as the percentage of Annexin V-PI double-positive cells. Macrophage viability was assessed by the calcein assay. Monocytes from the buffy coat were differentiated into macrophages with medium alone, CSF-1 or IL-34 and cell proliferation was analyzed at days 1, 3 and 7 by staining with Calcein-AM (1 μM, BD Bioscience) and analysis using a multi-label reader Victor3™ (PerkinElmer Inc.). Data were expressed as signal in arbitrary units.

### Real-time (RT) PCR and quantitative (q)PCR arrays

RNA from frozen synovial biopsies or from GM-CSF-, IL-34- and CSF-1-differentiated macrophages was isolated using the RNeasy Kit and RNase-Free DNase Set (Qiagen). Patient clinical characteristics are detailed in Additional file 1: Table S3. Total RNA was reverse-transcribed using SuperScript™ II RT (Invitrogen). Duplicate PCR reactions were performed using SYBR green (Applied Biosystems) with an ABI Prism® 7000 sequence detection system (Applied Biosystems). Complementary DNA (cDNA) was amplified using specific primers (Invitrogen) (see Additional file 1: Table S4). Relative levels of gene expression were normalized to *glyceraldehyde-3-phosphate dehydrogenase* (*GAPDH*) housekeeping gene. Messenger RNA (mRNA) expression was presented as 2^-ΔCt^ or relative quantity (RQ, 2^-ΔΔCt^). Alternatively, total RNA was subjected to cDNA synthesis using an RT^2^ First Strand Kit (Qiagen) and mRNA expression of 336 inflammation-related genes was analyzed by RT-qPCR using low density qPCR arrays (Human Angiogenic Growth Factors & Angiogenesis Inhibitors, Human Extracellular Matrix & Adhesion Molecules and Human TGFß/BMP Signaling Pathway PCR Arrays, Qiagen). Relative levels of gene expression were normalized to five housekeeping genes and RQ values determined as above.

### Antibodies

Anti-human CSF-1 Ab, clone 26730, a neutralizing mouse IgG2a monoclonal Ab, was purchased from R&D Systems, Minneapolis, MN, USA. Anti-human IL-34 Abs M2 and M4 were provided by Five Prime Therapeutics. M2 and M4 monoclonal Abs were prepared by immunization of Balb/c mice with purified human IL-34 produced at Five Prime Therapeutics, followed by standard hybridoma procedures and protein A purification. All animal experiments in this study were performed in compliance with appropriate protocols approved by the Institutional Animal Care and Use Committee at Five Prime Therapeutics and Bolder Biopath (Boulder, CO, USA). The M2 Ab was determined to be a blocking Ab, and the M4 Ab was determined to be a non-blocking Ab by testing hybridoma supernatant for the ability to block biotinylated IL-34-binding to human CSF1R-Fc in an ELISA format, and by testing the ability of purified Ab to neutralize IL-34-mediated survival of primary human monocytes. Anti-human CSF1R Ab huAB1, a humanized IgG4, monoclonal Ab, was provided by Five Prime Therapeutics. huAB1 blocks the binding of both CSF-1 and IL-34 to the human CSF1R, thereby inhibiting CSF1R-mediated signaling pathways (see Additional file 2: Figure S1a). Anti-mouse CSF1R Ab muAB5, was provided by Five Prime Therapeutics and is a chimeric IgG1, monoclonal Ab consisting of rat V regions and murine C regions. muAB5 blocks the binding of both mouse CSF-1 and mouse IL-34 to the mouse CSF1R, thereby inhibiting CSFR-mediated responses (see Additional file 2: Figure S1b). Anti-CSF1R treatment in mice has been reported to selectively reduce peripheral Ly6C^lo^ monocytes while having no effect on Ly6C^hi^ monocyte numbers [6, 18]. To assess the impact of anti-mouse CSF1R muAB5 on peripheral monocytes, we treated mice with 20 mg/kg of muAB5 or saline and 7 days later quantified monocyte populations in the blood. Anti-CSF1R muAB5 selectively reduced the number of Ly6Clo monocytes while having little to no effect on Ly6Chi monocytes (see Additional file 2: Figure S2).

### Synovial biopsy explant assays

Intact synovial biopsies (5 mm^3^) were obtained from the knees of patients with RA and were cultured, three biopsies per condition, for 24 h in complete DMEM supplemented with 10 % FCS and stimulated for 24 h with increasing concentrations of human IL-34 or CSF-1. Alternatively, explants were cultured for 96 h with anti-human CSF-1 (5 μg/ml), anti-IL-34 (5 μg/ml, M2), increasing concentrations of anti-human CSF1R Ab (huAB1), or their respective human isotype controls (Eureka Therapeutics). Cell-free tissue culture supernatants were harvested for cytokine analysis. Patient clinical characteristics are detailed in Additional file 1: Table S4.

### Measurement of cytokine production

Cell-free supernatants from synovial biopsies were analyzed by ELISA for IL-6 (PelKine Compact™ ELISA kits, Sanquin Reagents). CCL2, CCL-7, ENA-78, SDF-1, MIG, GCP-2, TAC, NAP-2, IP-10, IL-1β, TNF-α, CXCL-8, MMP-2, MMP-7, and MMP-9 were measured using human single-plex assays (Bio-Rad) and read on a Bio-Plex 200 system (Bio-Rad).

### Effects of anti-CSF1R Ab on murine myeloid homeostasis

CB17 SCID mice (3–5/group) were injected intravenously with 20 mg/kg of muAB5 or saline. Seven days later 50 μl of whole blood was harvested into FACS buffer containing ethylenediaminetetraacetic acid (EDTA) (MACs Rinsing Buffer with BSA; Miltenyi Biotech) to prevent clotting. Red blood cells were lysed by hypotonic lysis (0.16M NH_4_Cl, 0.01M KCO_3_ and 0.1mM EDTA). Samples were washed and Fc receptors blocked by staining with 5 μg/ml anti-CD16/32 Ab (Clone: 93, eBioscience). Cells were washed and stained with the following Abs: anti-mF4/80-FITC (Clone: BM/8; Biolegend), anti-mCD11b-PE (Clone: M1/70; BD Biosciences), anti-mLy6C-PerCP (Clone: HK1.4; Biolegend) and anti-mLy6G-Alexa700 (Clone: 1A8; Biolegend). To facilitate identification of dead cells the samples were stained with Aqua Live/Dead© (Invitrogen) according to the manufacturer’s recommendations. Cells were washed, pellets were resuspended in 50 μl of FACs buffer, and 50 μl of CountBright™ Absolute Counting Beads (Invitrogen) were added to each sample. Samples were thoroughly mixed and run on a BD LSRII flow cytometer (BD Biosciences). Data files were analyzed using Flow Jo software v.7.6.4 (Treestar Inc.)

### Induction and assessment of CIA and histopathological analysis

The mouse CIA models were established at Bolder Biopath. Male DBA/1 mice (12/group) were injected intradermally at the base of the tail with 150 μl of Freund’s Complete Adjuvant containing bovine type II collagen (2 mg/ml) on day 0 and day 21. For prophylactic treatment mice were dosed starting on day 0 with vehicle, Enbrel at 10 mg/kg, or muAB5 at 30 mg/kg. Treatment continued three times weekly through day 32. Clinical sores on a scale of 0–5 were determined for each of the paws on study days 18–35. The study was terminated on day 35 and blood was collected. Hind paws and knees were collected at termination and processed for histological analysis, as detailed in Additional file 3: Supplementary methods. For therapeutic treatment, mice were immunized as outlined above and randomized into treatment groups once swelling was obviously established in at least one paw. Group mean scores were 0.5–1.0 (out of a possible maximal score of 5.0) at the time of enrollment. Treatment with vehicle, Enbrel at 10 mg/kg, or muAB5 at 30 mg/kg was initiated after enrollment and continued three times weekly through day 20 of arthritis with mice terminated on day 23 of arthritis. Drug exposure as measured by the terminal plasma concentration was below the limit of detection in 3 out of 12 animals in the muAB5-treated group. These samples tested positive for anti-drug antibodies (data not shown), which likely contributed to the poor exposure. Based on these findings, these 3 animals with undetectable drug levels were excluded from any of the study analysis and interpretation: 7 the 12 animals in the Enbrel-treated group also had no detectable drug at termination and were excluded from the analysis.

### TRAP5b analysis

Mouse serum band 5 tartrate-resistant acid phosphatase isoform b (TRAP5b) was measured in mouse EDTA plasma using a commercial ELISA kit (MouseTRAP™ Assay, Immunodiagnostic Systems, Inc.) as per the manufacturer’s instructions, including a 1:4 dilution of all samples, using plasma instead of serum. Each sample was tested in singlet. The standard curve absorbance vs. concentration data were fit to a four-parameter logistic for calculation of the test results. Results are reported in units/liter (U/L).

### Statistical analyses

Statistical analysis was performed using Windows GraphPad Prism 5 (GraphPad Software, Inc.). Potential differences between experimental groups were analyzed by the non-parametric Mann-Whitney *U* test, Kruskal-Wallis test, or Friedman paired test, as appropriate. *P* values <0.05 were considered statistically significant.

## Results

### Synovial tissue expression of CSF-1, IL-34 and CSF1R

**Fig. 1:**
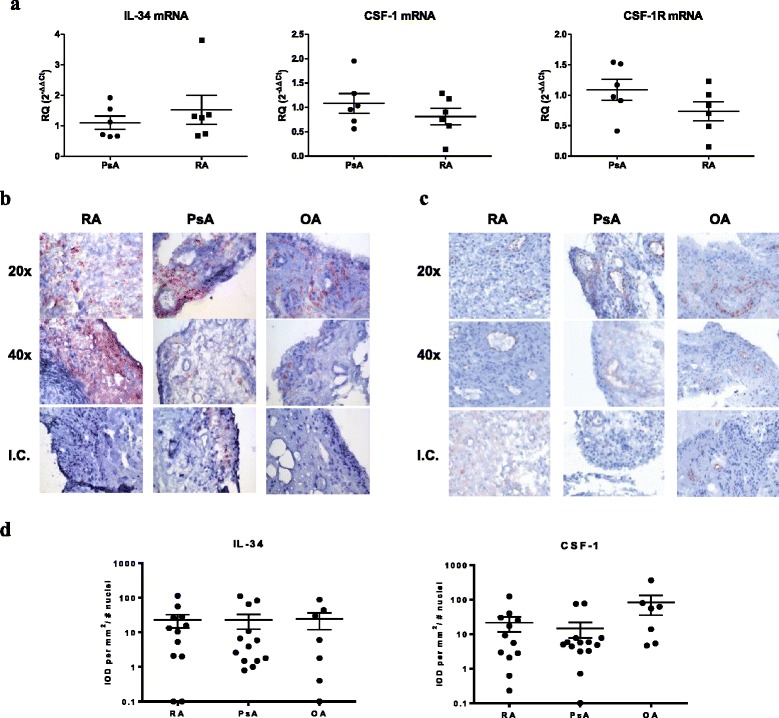
Colony-stimulating factor-1 (CSF-1), IL-34 and CSF1 receptor (*CSF1R*) are expressed in synovial tissue from patients with rheumatoid arthritis (*RA*) and psoriatic arthritis (*PsA*). **a** Quantification of relative IL-34, CSF-1 and CSF1R mRNA expression in synovial tissue from 6 patients with RA and 6 with PsA. Quantitative PCR data are shown as relative quantity (*RQ*), as described in “Methods”. Data are presented as scatter plots, where each *plot* represents an individual value, *bars* represent the mean, and *error bars* indicate the standard error of the mean (SEM). **b**, **c** Immunohistochemical analyses of RA, PsA and OA synovial tissue stained with control rabbit, anti-IL34 (**b**) and anti-CSF1 antibodies (**c**). **d** Quantitative analysis of IL-34 and CSF-1 staining in synovial tissue. Synovial sections from 15 patients with RA, 15 with PsA and 7 patients with osteoarthritis (*OA*) were stained with antibodies against IL-34 and CSF-1 antibodies as above, and the integrated optical density (*IOD/mm* ^*2*^) corrected for cellularity was calculated by digital image analysis. Each *plot* represents an individual value, *bars* represent the mean, and *error bars* indicate the SEM. *P < 0.05

First, we investigated the expression of CSF-1, IL-34 and CSF1R in the synovial tissue of patients with RA or PsA. qPCR analysis did not identify any differences between RA and PsA patients in mRNA expression of IL-34, CSF-1, or their receptors (Fig. 1a). Immunohistochemical analysis of synovial tissue independently confirmed that IL-34 (Fig. 1b) and CSF-1 (Fig. 1c) are expressed in synovial tissue in RA, PsA and OA. While IL-34 is expressed in the synovial sublining and the intimal lining layer, CSF-1 expression was limited primarily to the areas surrounding the blood vessels. On digital quantification of staining for each cytokine, IL-34 and CSF-1 protein expression was similar in synovial tissue in RA, PsA, and OA (Fig. 1d). Together, these data demonstrate that both IL-34 and CSF-1 are expressed at similar levels in the synovium of patients with inflammatory and non-inflammatory arthritis.

### IL-34 and CSF-1 induce similar but distinct gene expression patterns in human macrophages

**Fig. 2:**
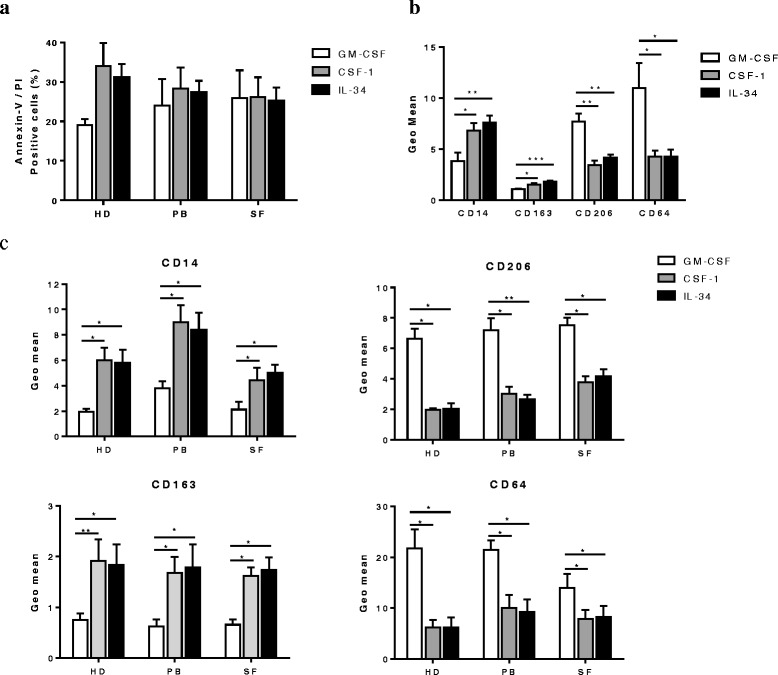
IL-34 and colony stimulating factor-1 (*CSF-1*) macrophages have similar phenotypic characteristics. **a** Fluorescence-activated cell sorting (FACS) analysis of Annexin V-propidium iodide (*PI*) staining in macrophages differentiated for 7 days in granulocyte-macrophage colony-stimulating factor (*GM-CSF*) (5 ng/ml), CSF-1 (25 ng/ml) or IL-34 (25 ng/ml) from peripheral blood and synovial fluid of healthy donors (*HD*) and patients with rheumatoid arthritis (RA). Data are presented as percentage of double-positive cells and represent the mean ± standard error of the mean (SEM) of 3–4 independent experiments. **b**, **c** FACS analysis of expression of macrophage surface markers CD14, CD163, CD206, and CD64 in macrophages differentiated for 7 days in GM-CSF, CSF-1 or IL-34 derived from monocytes of buffy coat (**b**) or monocytes from peripheral blood of HD or patients with RA (*PB*), or from synovial fluid from patients with RA (*SF*) (**c**). Data are presented as the geometric mean (*geo mean*) and represent the mean ± SEM of 4–5 independent experiments per marker. *P < 0.05, **P < 0.01, and ***P < 0.001

Given that IL-34 and CSF-1 localized to distinct regions of the synovium, and macrophages in RA and PsA synovial sublining and intimal lining layers display distinct phenotypic characteristics [43], we examined the influence of these two cytokines in peripheral blood from HD and patients with RA, and their influence on synovial fluid-derived monocyte differentiation, proliferation and gene expression in RA. To test this, we differentiated monocytes from mononuclear cells of buffy coats in blood from HD, and peripheral blood and synovial fluid from patients with RA with CSF-1 (CSF-1 Mφ), IL-34 (IL-34 Mφ) or GM-CSF (GM-CSF Mφ). We first analyzed the effect of IL-34 and CSF-1 on macrophage proliferation and survival. We observed no differences in cell survival in IL-34 Mφ and CSF-1 Mφ; however, there was a trend towards greater cell death compared to GM-CSF Mφ (Fig. 2a). We did not observe any differences in cell viability between macrophages derived from mononuclear cells in peripheral blood from HD or patients with RA, nor between peripheral blood-derived and synovial fluid-derived macrophages in patients with RA (Fig. 2a). We also found that both CSF-1 and IL-34 promoted cell survival similarly, and significantly enhanced viability compared to macrophages differentiated in medium alone (*p* < 0.05, see Additional file 2: Figure S3).

Next, we examined the expression of recently validated polarization markers for human macrophages [43]. We observed that the expression of CD14, CD163, CD206, and CD64 was similar in IL-34 Mφ and CSF-1 Mφ, independently if the macrophages were derived from peripheral blood from HD, or peripheral blood or synovial fluid from patients with RA. In contrast, GM-CSF Mφ had significantly lower expression of CD14 and CD163, and elevated expression of CD206 and CD64 (Fig. 2b and Fig. 2c).

**Fig. 3:**
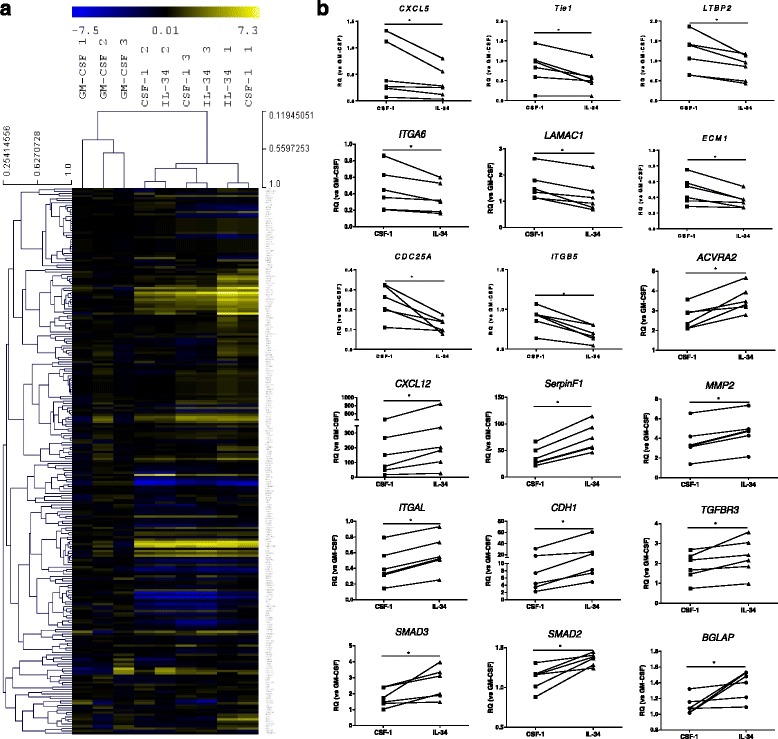
Gene expression in differentiated macrophages. **a** mRNA expression profiles of 336 genes involved in angiogenesis, extracellular matrix remodeling, and osteoclast formation in granulocyte-macrophage colony-stimulating factor (*GM-CSF*)-, colony-stimulating factor-1 (*CSF-1*)- or IL-34-differentiated macrophages (Mφ) derived from monocytes of buffy coats (n = 3). Data are presented in an unsupervised clustergram. **b** Analyses of mRNA expression levels of selected genes analyzed in **a**. Data are shown as relative quantity (*RQ*) in relation to GM-CSF Mφ, as described in “Methods”

**Fig. 4:**
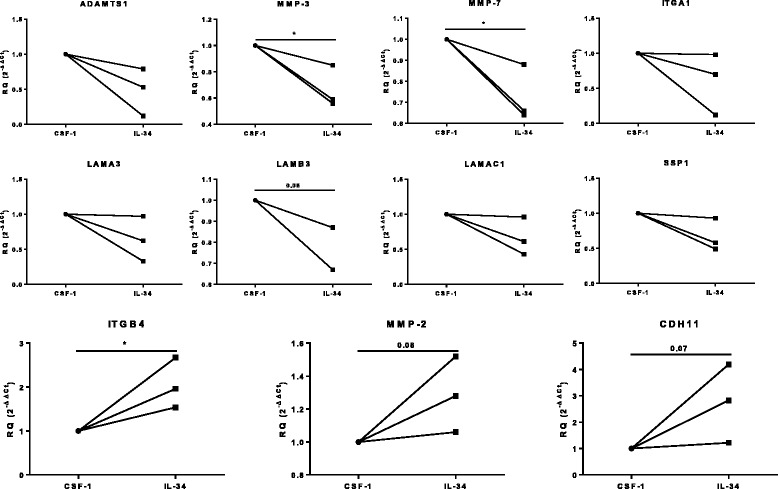
Gene expression in macrophages differentiated from synovial fluid monocytes in rheumatoid arthritis (RA). Analyses of mRNA expression levels of genes involved in extracellular matrix remodeling formation in colony-stimulating factor-1 (*CSF-1*)- or IL-34-differentiated macrophages (Mφ) derived from monocytes of synovial fluid in RA (n = 3). Data are shown as relative quantity (*RQ*) in CSF-1 Mφ, as described in “Methods”. *P < 0.05

Finally, we examined the mRNA expression of 336 genes relevant to RA, involved in angiogenesis, extracellular matrix remodeling and osteoclast formation. On unsupervised clustergram analysis, GM-CSF Mφ derived from peripheral blood from HD clustered distinctly compared to those differentiated in IL-34 or CSF-1 (Fig. 3a), as expected. IL-34 and CSF-1 Mφ were closely related, with more global differences between monocyte donors than the cytokine used for differentiation. However, despite the strong relationship in gene expression between macrophages differentiated in IL-34 and CSF-1, we identified a number of genes that were significantly upregulated in CSF-1 Mφ, including *CXCL5*, *TIE1*, *LMBP2*, *CDC25A*, *ITGA6*, *LAMAC1*, *ECM1*, and *ITGB5* (Fig. 3a). In contrast, the expression of *CXCL12*, *SERPINF1*, *MMP2*, *ACVRA2*, *ITGAL*, *CDH11*, and *TGFBR3* was significantly upregulated in IL-34 Mφ (Fig. 3b and Additional file 1: Table S5). We also examined whether IL-34 and CSF-1 Mφ differentiated from synovial fluid (SF) monocytes in RA had different expression patterns in genes related to extracellular matrix remodeling. We observed upregulation of *ADAMTS1*, *MMP3*, *MMP7*, *ITGA1*, *LAMA1*, *LAMA3*, *LAMB3*, and *SSP1* in CSF-1 Mφ, while *CDH11*, *MMP2*, and *ITGB4* were upregulated in IL-34 Mφ (Fig. 4). Together, these results suggest that while IL-34 and CSF-1 generate phenotypically similar macrophages, differential localized production of IL-34 and CSF-1 in the synovium could potentially give rise to macrophages with discrete functional capacities.

### CSF1R blockade reduces inflammatory mediator production in RA synovial tissue

**Fig. 5:**
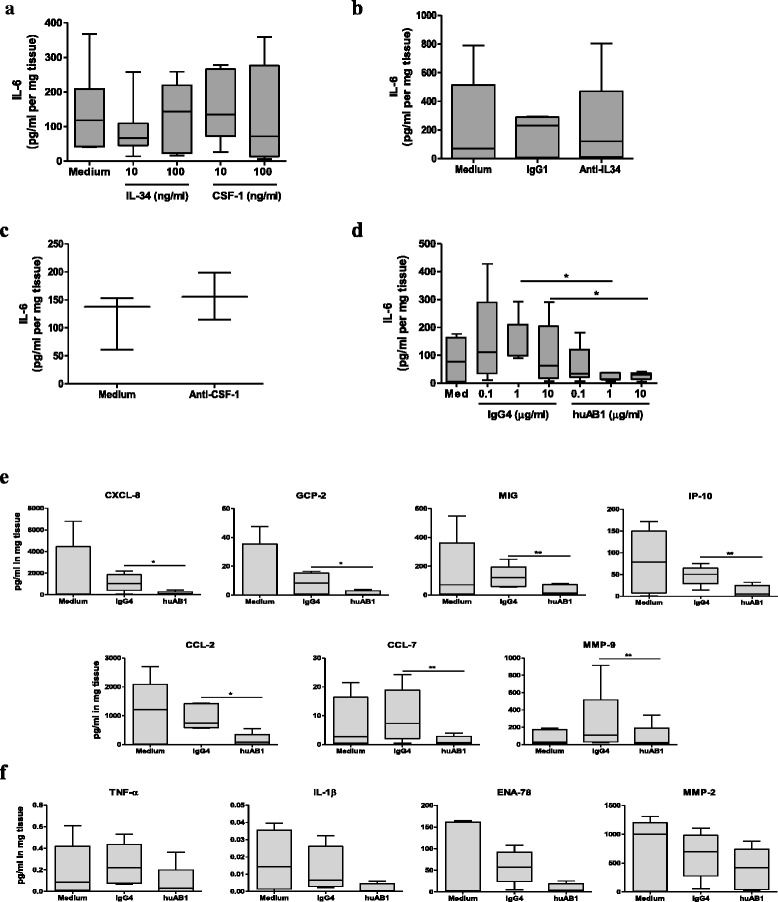
Anti-colony stimulating factor-1 receptor (anti-CSFR1) antibody reduces the production of inflammatory mediators in synovial tissue in rheumatoid arthritis (RA). **a** IL-6 ELISA production in supernatants of RA synovial tissue after 24-h incubation in medium alone or increasing concentrations of colony stimulating factor-1 (*CSF-1*) or IL-34 (n = 7). **b**-**d** IL-6 ELISA production in supernatants of RA synovial tissue after 4 days of incubation in medium alone in the presence of IgG1 or anti-IL-34 antibody (Ab) (5 μg/ml for both, n = 5) (**b**), anti-CSF-1 Ab (5 μg/ml, n = 3) (**c**), or increasing concentrations of IgG4 or huAB1 (n = 5) (**d**). **e**, **f** Multiplex analysis of protein production in supernatants of RA synovial tissue after 4 days of incubation in medium alone in the presence of 1 μg/ml IgG4 or 1 μg/ml huAB1 (n = 4). *Boxes* represent the 25th–75th percentiles, *lines within the boxes* mark the median value, and *lines outside the boxes* denote the 10th and 90th percentiles. *P < 0.05 and **P < 0.01

To extend our knowledge about the role of CSF1R signaling in RA, we used blocking antibodies to CSF-1 (clone 26730), to IL-34 (M2), or to CSF1R (huMab1) to investigate the differential effects of inhibiting responses to each ligand independently versus inhibiting responses to both ligands simultaneously through CSF1R blockade. We first determined the effect of IL-34 and CSF-1 stimulation on cytokine production in RA synovial tissue. Initial experiments demonstrated that addition of either exogenous IL-34 or CSF-1 did not induce IL-6 production by RA synovial explants (Fig. 5a). To discard the possibility that the receptor-type protein-tyrosine phosphatase ζ (PTP-ζ), a recently discovered IL-34 receptor [44], could interfere in IL-6 production, we analyzed the mRNA expression in the synovial tissue of patients with RA or PsA. We found that PTP-ζ was expressed in the synovium in both diseases (see Additional file 2: Figure S4), albeit at very low levels compared to CSF1R. Moreover, neutralization of either IL-34 alone (Fig. 5b) or of CSF-1 alone (Fig. 5c) did not influence IL-6 production by synovial explants. However, direct targeting of CSF1R with huAb1, which blocks binding of both IL-34 and CSF-1, significantly reduced synovial explant IL-6 production in a dose-dependent manner, with maximum inhibition already observed at an Ab concentration of 1 μg/ml (Fig. 5d). huAB1 also significantly reduced production of the chemokines CXCL-8, GCP-2, MIG, IP-10, CCL-2, CCL-7, and MMP-9 in RA synovial tissue (Fig. 5e), and we observed a trend towards a reduction in production of TNF, IL-1β, ENA-78 and MMP-2, although the differences were not significant (Fig. 5f). In contrast, we did not observe differences in the production of SDF-1, TAC, NAP-2, and MMP-7 (data not shown). Together, these results demonstrate that CSF1R signaling inhibition, but not individual inhibition of the two receptor ligands, is able to reduce the production of inflammatory mediators in synovial tissue in RA.

### CSF1R blockade is protective in CIA

**Fig. 6:**
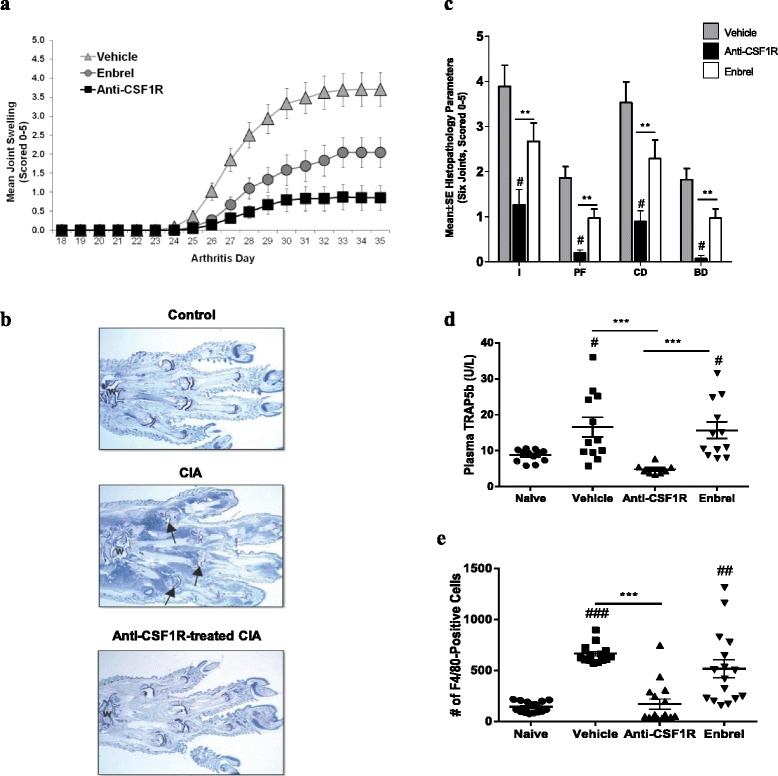
Prophylactic treatment with anti-colony stimulating factor-1 receptor (*anti-CSF1R*) prevents pathological progression in collagen-induced arthritis (*CIA*). **a** Daily global arthritic scores for mice treated intraperitoneally every 3 days with vehicle, Enbrel, or muAb5. Treatment continued three times weekly from day 0 with control vehicle (*triangles*), Enbrel (10 mg/kg) (*circles*) or muAb5 (30 mg/kg) (*squares*). **b** Representative images of pathological appearances of the joints in the indicated treatment group, visualized by H&E staining. Note the representative areas of synovial cellular infiltration and pannus formation (*arrows*). **c** Inflammation (*I*), pannus formation (*PF*), cartilage damage (*CD*), and bone damage (*BD*) scores for mice in each treatment group. Data are mean ± standard error of the mean (SEM) for each group (n = 12 animals per group). ^#^ *P* < 0.05 vs vehicle and **P < 0.01. **d**, **e** Band 5 tartrate-resistant acid phosphatase isoform b (*TRAP5b*) plasma levels (**d**) and number of F4/80-positive cells in five × 200 fields (**e**) in the inflamed joints of mice in each treatment group. *Symbols* represent values obtained from individual animals, *bars* represent the mean, and *error bars* indicate the SEM. ^#^ *P* < 0.05 vs naive and ^###^ *P* < 0.001 vs naïve. ***P < 0.001

Given the efficacy of CSF1R blockade in RA synovial tissue, we examined the effect of the blocking anti-mouse CSF1R antibody muAB5 in vivo using the murine CIA model of arthritis. Prophylactic administration of muAB5 (30 mg/kg) significantly reduced the severity of the arthritis compared with the vehicle control (Fig. 6a). Histological analyses of the hind paws of the mice confirmed the protective effect of muAB5, as the scores for inflammation, cartilage damage, pannus formation, and bone erosion were all improved in muAB5-treated mice (Fig. 6b). Semiquantitative analysis demonstrated that muAB5 administration drastically and significantly reduced all these analyzed parameters (Fig. 6c). As CSF1R signaling also supports osteoclastogenesis, we quantified the serum levels of the bone resorption marker, TRAP5b. Anti-CSF1R-treated mice had a significant reduction in TRAP5b compared with vehicle-treated mice (Fig. 6d). Interestingly, administration of muAB5 was more effective than Enbrel (10 mg/kg) in reducing the severity of arthritis and in improvement of all histological, radiological, and serological parameters (Fig. 6a-d). Anti-CSF1R treatment was associated with reduced macrophage infiltration in the synovial tissue (Fig. 6e). The front paws and knees of the mice were examined by immunohistochemical analysis for the presence of macrophages by F4/80 staining. Macrophage numbers were significantly reduced in muAB5-treated mice compared to vehicle control. Enbrel had no significant effect on tissue macrophage numbers.

**Fig. 7:**
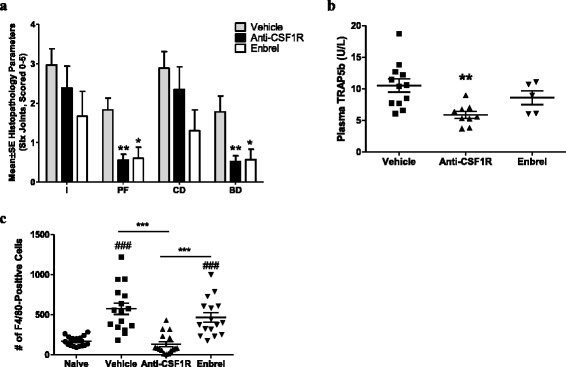
Therapeutic treatment with anti-colony stimulating factor-1 receptor (*anti-CSF1R*) suppresses bone destruction and pannus formation in collagen-induced arthritis (*CIA*). **a** Histopathological assessment of CIA in mice treated with vehicle, Enbrel, or muAB5 after the initiation of arthritis. *P < 0.05 and **P < 0.01 vs vehicle. *I* inflammation, *PF* pannus formation, *CD* cartilage damage, *BD* bone damage. **b**, **c** Band 5 tartrate-resistant acid phosphatase isoform b (*TRAP5b*) plasma levels (**b**) and number of F4/80-positive cells in five × 200 fields (**c**) in the inflamed joints of mice in each treatment group. *Symbols* represent values obtained from individual animals, *bars* represent the mean, and *error bars* indicate the standard error of the mean. **P < 0.01 vs vehicle and ^###^ *P* < 0.001 vs naïve. ***P < 0.001

We next examined the therapeutic effect of muAB5 treatment in the CIA model. muAB5 administered after the induction of arthritis significantly inhibited pannus formation and bone destruction (Fig. 7a). Anti-CSF1R treatment did not have a significant effect on histological parameters of inflammation or cartilage destruction. In this study Enbrel also had no significant effect on inflammation or cartilage destruction. Therapeutic treatment with muAB5 also reduced serum TRAP5b and the number of tissue macrophages (Fig.7b and 7c). The lack of an effect of muAB5 on inflammation, despite reducing tissue macrophage numbers, is possibly due to the fact that late-stage inflammation in the mouse CIA model is driven in part by high infiltration of neutrophils. This is in contrast to human RA in which macrophages predominate.

## Discussion

In the present study we demonstrated that specific antibodies against CSF1R, which prevent binding of both CSF-1 and IL-34 to their receptor, reduce the severity of CIA and the production of inflammatory mediators in RA synovial tissue explants ex vivo. Conversely, neutralization of CSF-1 or IL-34 individually had no detectable effect on inflammatory gene expression in RA synovial tissue. This latter observation was surprising, as CSF-1 deficiency or neutralization in animal models of RA has clear therapeutic effects [11, 12]. Our data might suggest that in CIA, CSF-1 plays an important role in promoting the differentiation and survival of mature monocytes in the periphery before or as they are entering the inflamed tissue, as observed in certain murine models of lung and peritoneal inflammation [6], while in RA synovial explants, synovial macrophages are no longer dependent upon CSF-1 alone. Instead, synovial macrophages may also utilize IL-34 as a survival factor. In line with this, CSF-1 and IL-34 are detected at equivalent concentrations in synovial fluid from patients with RA, and here we confirm and extend previous observations that both CSF-1 and IL-34 are produced locally in RA synovial tissue [35, 36]. However, stimulation of RA synovial biopsies with either CSF-1 or IL-34 failed to influence acute expression of inflammatory mediators, possibly indicating that endogenous synovial production of CSF-1 and/or IL-34 in RA is saturating in regard to CSF1R availability.

Our inability to modulate RA synovial explant gene expression by neutralizing CSF-1 and IL-34 individually further supports the notion that these two cytokines are largely redundant in supporting the differentiation and survival of most tissue macrophage populations. Some subtle differences have been noted between CSF-1 and IL-34 in the strength and kinetics with which they activate CSFR1 [30–32]. Indeed, global gene expression analysis of human monocytes differentiated with IL-34 and M-CSF revealed that of the genes regulated by these cytokines, quantitative differences in the induction were noted for approximately 30 % of the genes [45]. Consistent with this, of 336 genes involved in processes contributing to pathological changes in RA, such as angiogenesis, inflammation, and tissue remodeling, we observed only 18 genes that were differentially expressed by CSF-1 and IL-34 Mφ. However, as we observe that CSF-1 (detected in cells surrounding the blood vessels) and IL-34 (throughout the synovial sublining and intimal lining layers) are expressed in different regions of the synovium, these cytokines could potentially contribute to the phenotypic heterogeneity of macrophages observed in the sublining and intimal lining layers of synovium in inflammatory arthritis [46].

Our results provide the first direct evidence that targeting of CSF1R has anti-inflammatory effects in not only animal, but also human models of established RA. Previous studies have observed protective effects against pathological changes in animal models of RA using pharmacological inhibitors of CSF1R kinase activity [22, 23]. However, concerns have been raised that these compounds can also target other tyrosine kinases relevant to pathological change in RA [17]. While this manuscript was in preparation, Toh and colleagues reported that another CSF1R-specific Ab could prophylactically prevent inflammation and joint damage in CIA [47]. Intriguingly, the same antibody did not suppress inflammation in the murine serum transfer model of arthritis, while retaining its capacity to block cartilage and bone destruction. The authors attributed this discrepancy to potentially differential roles for macrophage recruitment and activation in these animal models [47].

Similarly, conflicting results using different CSF1R-blocking antibodies in other animal disease models have led to questions about the feasibility of this strategy [17]. For instance, the anti-CSFR1 Ab AFS98, which rapidly reduces monocytes and tissue macrophages in vivo, confers protection in murine models of lung and peritoneal inflammation [6]. However, another Ab M279, which reduces the F4/80^hi^Ly6^lo^ monocyte population and tissue macrophages more slowly, does not display anti-inflammatory effects in similar models, but exacerbates graft-versus-host disease [18]. The difference in activity of AFS98 and M279 could be due to differences in Fc effector function [17]. AFS98 is a rat IgG2a with greater potential for Fc effector function compared to M279, which is a rat IgG1. muAb5 is a mouse IgG1 and expected to have low Fc effector function, similar to M279. In our study, muAb5 reduced the same circulating monocyte population observed with M279, but provided almost complete protection against all parameters of disease in CIA.

These studies have led to the suggestion that in some inflammatory diseases, anti-CSF1R therapy might aggravate pathological changes by depleting the tissue of the tolerizing resident tissue macrophages and allowing their replacement by pro-inflammatory monocyte populations, or by promoting pro-inflammatory re-polarization of tissue macrophages [17]. These concerns can only be addressed empirically in the clinic, but it is noteworthy that huAB1 treatment reduced the production of pro-inflammatory cytokines (IL-6, TNF, and IL-1β), chemokines (CXCL-8, GCP-2, MIG, IP-10, CCL-2, and CCL7), and MMPs (MMP-2 and MMP-9) in RA synovial tissue. This suggests that CSF1R blockade in the inflamed tissue does not result in conversion of synovial macrophages to a more pro-inflammatory functional phenotype, consistent with recent observations that environmental stimuli present in synovial tissue, such as IgG complexes and angiopoietins, can override polarization conditions to regulate macrophage gene expression [48, 49]. Experimentation in synovial biopsies cannot address the possibility that circulating pro-inflammatory monocytes might replace synovial macrophages following anti-CSF1R treatment, but studies of monocyte migration using scintigraphy in RA patients has indicated that macrophage turnover in synovial tissue is slow, and even during successful therapy, the rate of monocyte immigration into synovial tissue is unchanged [50, 51], raising the possibility that CSF-1 maintains distinct monocyte populations in mouse and man.

## Conclusions

Simultaneous interference with CSF-1 and IL-34 signaling to CSF1R suppresses pro-inflammatory gene expression in RA synovial tissue, and decreases pathology in both prophylactic and therapeutic treatment strategies in CIA, further validating CSF1R as a potential therapeutic target in RA.

## Additional files

Additional file 1: Supplementary Tables.
**Table S1**. Clinical characteristics of patients used for immunohistochemistry analysis. **Table S2**. Clinical characteristics of patients used for qPCR analysis. **Table S3**. Clinical characteristics of patients used for experiments with synovial biopsy explants. **Table S4**. List of primers used for qPCR analysis. **Table S5**. Comparison of expression of 84 genes involved in the regulation of angiogenic processes (A), extracellular matrix remodeling (B) and TGF/BMP signaling (C) in macrophages differentiated in CSF-1 or IL-34 for 7 days. Results indicate the RQ in relation to GM-CSF macrophages, as described in “Methods”, and are presented as the mean of six independent experiments. (DOC 126 kb) Additional file 2: Supplementary Figures.
**Figure S1**. huAB1 and muAB5 antibodies block ligand-induced CSFR1 responses. **a** CSFR1 phosphorylation assay in IL-34 and CSF-1 stimulated CHO-CSF1R cells in the presence of increasing concentrations of huAB1. **b** Monocyte proliferation in IL-34- and CSF-1-stimulated NFS60 cells in the presence of increasing concentrations of muAB5. **Figure S2**. Circulating Ly6Clo monocyte numbers are reduced following treatment with muAB5. Graphs show the absolute number of **a** LyC6lo and **b** Ly6Chi monocytes present in blood samples obtained from muAB5- or saline-treated mice. *Symbols* represent values obtained from individual animals, *bars* represent the mean and *error bars* indicate the SEM. **P* value <0.05; Mann-Whitney *U* test. **Figure S3**. IL-34 and CSF-1 macrophages have similar viability. Cell viability assay of monocytes from buffy coat differentiated in medium, CSF-1 or IL-34 for 1, 3 and 7 days. Data are presented as arbitrary units and represent the mean ± SEM of four independent experiments. **P* < 0.05 compared to medium macrophages. **Figure S4**. PTP-ζ mRNA expression in synovial tissue from patients with rheumatoid arthritis (*RA*) or psoriatic arthritis (*PsA*) is almost residual compared to CSF-1R. Quantification of relative PTP-ζ and CSF1R mRNA expression in synovial tissue from 6 patients with RA and 6 patients with PsA. qPCR data are shown as 2 ^-ΔCt^, as described in “Methods”. Data are presented as scatter plots, where each *plot* represents an individual value, *bars* represent the mean and error bars indicate the SEM. (PDF 146 kb) Additional file 3:
**Supplementary methods**. (DOCX 16 kb)

## Abbreviations

Ab: antibody
CIA: collagen-induced arthritis
cDNA: complementary DNA
CSF: colony-stimulating factor
CSF1R: colony - stimulating factor-1 receptor
DMEM: Dulbecco’s modified Eagle’s medium
EDTA: ethylenediaminetetraacetic acid
ELISA: enzyme-linked immunosorbent assay
FACS: fluorescence-activated cell sorting
FCS: fetal calf serum
FLS: fibroblast-like synoviocyte
GAPDH: glyceraldehyde-3-phosphate dehydrogenase
GM-CSF: granulocyte-macrophage colony-stimulating factor
HD: healthy donor
HRP: horseradish peroxidase
H&E: hematoxylin and eosin
IOD: integrated optical densities
IL: interleukin
MMP: matrix metalloproteinase
mRNA: messenger RNA
OA: osteoarthritis
PI: propidium iodide
PsA: psoriatic arthritis
RA: rheumatoid arthritis
RQ: relative quantity
RT-qPCR: real-time quantitative polymerase chain reaction
SF: synovial fluid
TNF: tumor necrosis factor
TRAP5b: band 5 tartrate-resistant acid phosphatase isoform b
