# Supplementary material for: Colony-stimulating factor (CSF) 1 receptor blockade reduces inflammation in human and murine models of rheumatoid arthritis
Source: Arthritis Res Ther. 2016 Mar 31;18:75. doi: 10.1186/s13075-016-0973-6 (PMC4818474; doi:10.1186/s13075-016-0973-6)
Supplement: Additional file 1: Supplementary Tables. — Table S1. Clinical characteristics of patients used for immunohistochemistry analysis. Table S2. Clinical characteristics of patients used for qPCR analysis. Table S3. Clinical characteristics of patients used for experiments with synovial biopsy explants. Table S4. List of primers used for qPCR analysis. Table S5. Comparison of expression of 84 genes involved in the regulation of angiogenic processes (A), extracellular matrix remodeling (B) and TGF/BMP signaling (C) in macrophages differentiated in CSF-1 or IL-34 for 7 days. Results indicate the RQ in relation to GM-CSF macrophages, as described in “Methods”, and are presented as the mean of six independent experiments. (DOC 126 kb) [file 13075_2016_973_MOESM1_ESM.doc]

**Table S1. Clinical characteristics of patients used for immunohistochemistry analysis.**

| **Characteristic** | **RA (n=15)** | **PsA (n=15)** | **OA (n=7)** |
| --- | --- | --- | --- |
| **Age (y): median (IQR)** | 43 (34-58) | 49 (40-56) | 65 (51-73) |
| **Female:male (n/n)** | 13/2 | 6/9 | 3/4 |
| **Disease duration (months): median (IQR)** | 6 (1-9) | 5 (0-15) | 2 (1-12) |
| **RF positive: n/total (%)** | 10/15 (67) | 0/15 (0) | 1/7 (14) |
| **ACPA positive: n/total (%)** | 9/15 (60) | 0/15 (0) | 0/7 (0) |
| **ESR (mm/h): median (IQR)** | 29 (7.7-59.7) | 23 (5-32) | 5 (0/9) |
| **CRP (mg/l): median (IQR)** | 14.9 (3-50.9) | 6.1 (2.3-14.7) | 1 (0-1.4) |
| **DAS28: median (IQR)** | 5.5 (4.2-6.1) | 5 (3.9-5.6) | 1.6 (0-3.1) |
| **Receiving MTX: n/total (%)** | 6/15 (40) | 1/15 (6) | 0/7 (0) |
| **Corticosteroids: n/total (%)** | 4/15 (27) | 0/15 (0) | 1/7 (14) |
| **Anti-TNF: n/total (%)** | 2/15 (13) | 0/15 (0) | 0/7 (0) |
| **Other biologic drug: n/total (%)** | 0/15 (0) | 0/15 (0) | 0/7 (0) |

ESR = erythrocyte sedimentation rate; CRP = C-reactive protein; DAS28 = disease activity score 28, RF=rheumatoid factor, ACPA=anti-cyclic citrullinated peptide antibody; MTX=methotrexate

**Table S2. Clinical characteristics of patients used for qPCR analysis.**

| **Characteristic** | **RA (n=6)** | **PsA (n=6)** |
| --- | --- | --- |
| **Age (y): median (IQR)** | 62 (60-63) | 50 (39-62) |
| **Female:male (n/n)** | 4/2 | 4/2 |
| **Disease duration (months): median (IQR)** | 127 (53-159) | 28 (16-41) |
| **RF positive: n/total (%)** | 4/5 (80%) | N/A |
| **ACPA positive: n/total (%)** | 4/5 (80%) | N/A |
| **ESR (mm/h): median (IQR)** | 17 (15-40) | 12 (11-37) |
| **CRP (mg/l): median (IQR)** | 7.7 (4.0-17.0) | 3.9 (2.7-4.7) |
| **DAS28: median (IQR)** | 4.57 (4.14-6.94) | 4.21 (3.58-4.73) |
| **Receiving MTX: n/total (%)** | 5/6 (83) | 2/6 (33) |
| **Corticosteroids: n/total (%)** | 4/6 (67) | 1/6 (17) |
| **Anti-TNF: n/total (%)** | 2/6 (33) | 1/6 (17) |
| **Other biologic drug: n/total (%)** | 1/6 (17) | 0/6 (17) |

ESR = erythrocyte sedimentation rate; CRP = C-reactive protein; DAS28 = disease activity score 28, RF=rheumatoid factor, ACPA=anti-cyclic citrullinated peptide antibody; MTX=methotrexate

**Table S3.** Clinical characteristics of patients used for experiments with synovial biopsy explants.

| **Characteristic** | **RA (n=10)** |
| --- | --- |
| **Age (y): median (IQR)** | 63 (57-67) |
| **Female:male (n/n)** | 8/2 |
| **Disease duration (months): median (IQR)** | 129 (105-272) |
| **RF positive: n/total (%)** | 7/10 (70) |
| **ACPA positive: n/total (%)** | 7/10 (70) |
| **ESR (mm/h): median (IQR)** | 22 (13-35) |
| **CRP (mg/l): median (IQR)** | 6.1 (3.3-57.2) |
| **DAS28: median (IQR)** | 3.83 (3.2-4.3) |
| **Receiving MTX: n/total (%)** | 9/10 (90) |
| **Corticosteroids: n/total (%)** | 5/10 (50) |
| **Anti-TNF: n/total (%)** | 2/10 (20) |
| **Other biologic drug: n/total (%)** | 2/10 (20) |

ESR = erythrocyte sedimentation rate; CRP = C-reactive protein; DAS28 = disease activity score 28, RF=rheumatoid factor, ACPA=anti-cyclic citrullinated peptide antibody; MTX=methotrexate

**Table S4.** List of primers used for qPCR analysis.

| **Gene** | **Primer forward** | **Primer reverse** |
| --- | --- | --- |
| **M-CSF** | 5’GTTTGTAGACCAGGAACAGTTGAA3’ | 5’CGCATGGTGTCCTCCATTAT3’ |
| **IL-34** | 5’GTCCTTAGGCCTCTGTGGAC3’ | 5’GCCAAGGAAGATCCCAAGATA3’ |
| **CSF-1R** | 5’GTGGCTGTGAAGATGCTGAA3’ | 5’CCTTCCTTCGCAGAAAGTTG3’ |
| PTP-ζ | 5’ATTCTGCAGCCCTAAAGCAA3’ | 5’AGGAGAGGGTGCTGGGTAAT3’ |
| **GAPDH** | 5’GCCAGCCGAGCCACATC3’ | 5’TGACCAGGCGCCCAATAC3’ |

**Table S5.** Comparison of expression of 84 genes involved in the regulation of angiogenic processes (A), extracellular matrix remodeling (B) and TGF/BMP signaling (C) in macrophages differentiated in CSF-1 or IL-34 for 7 days. Results indicate the RQ in relation to GM-CSF macrophages, as described in materials and methods, and are presented as the mean of 6 independent experiments.
