# Supplementary figures and images for: Colony-stimulating factor (CSF) 1 receptor blockade reduces inflammation in human and murine models of rheumatoid arthritis
Source: Arthritis Res Ther. 2016 Mar 31;18:75. doi: 10.1186/s13075-016-0973-6 (PMC4818474; doi:10.1186/s13075-016-0973-6)

Figure S1

a

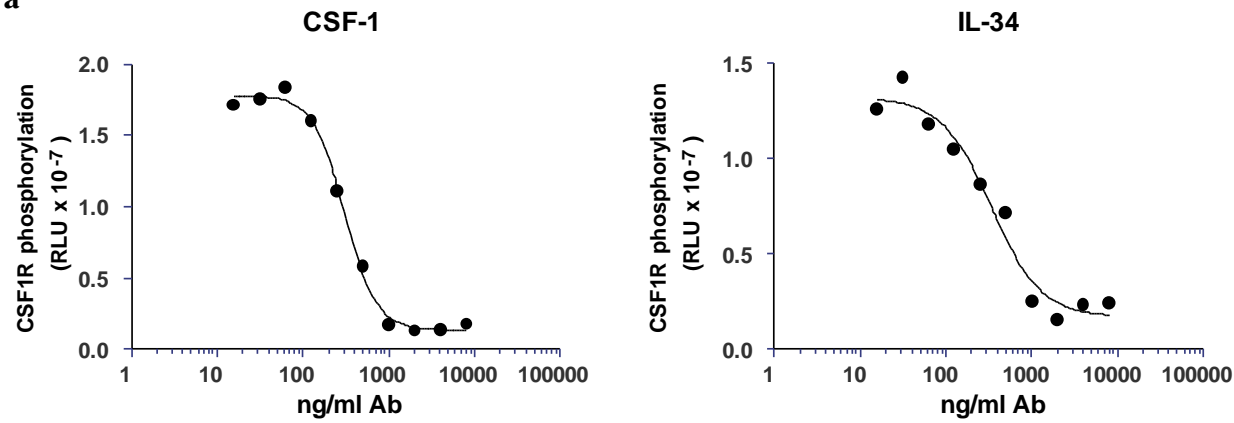

b

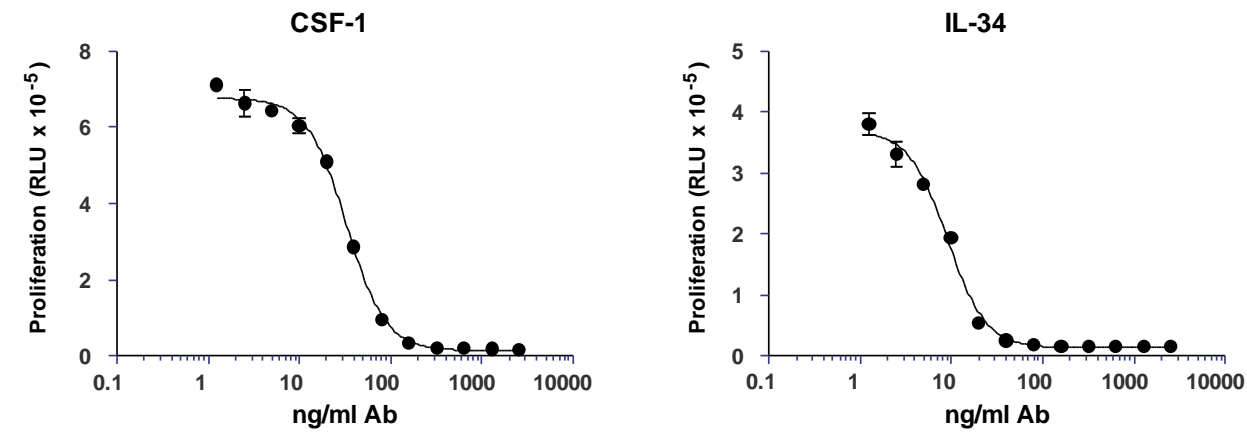

Figure S2

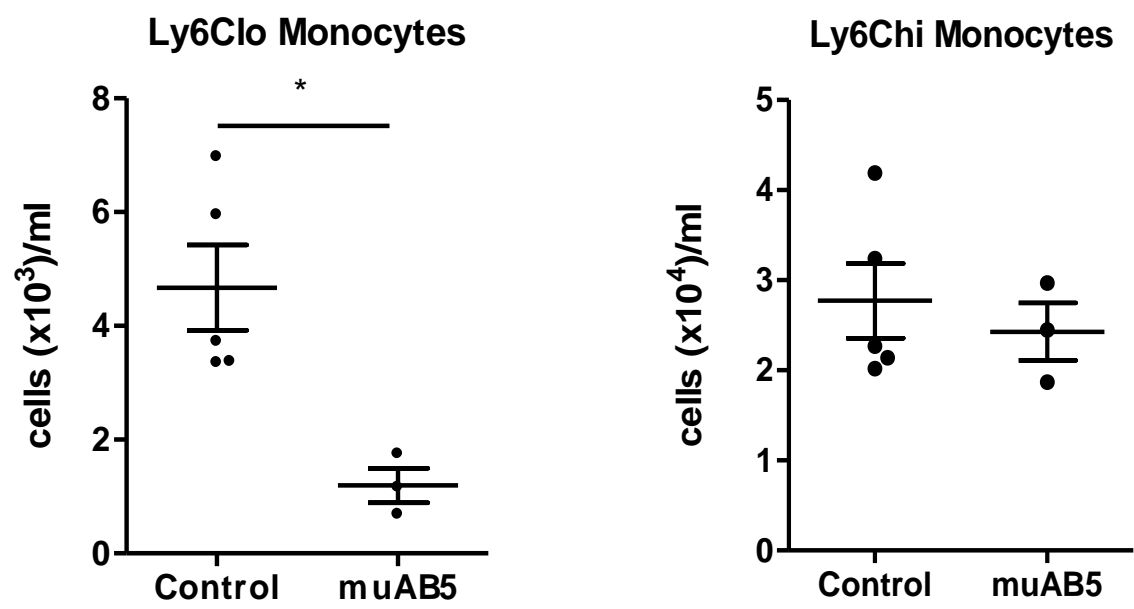

Figure S3

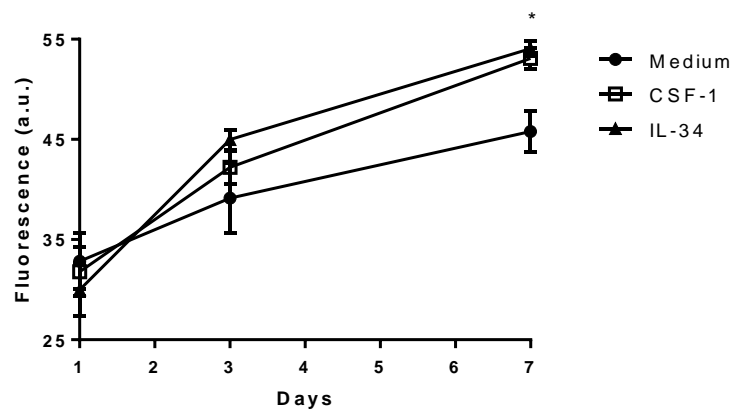

Figure S4

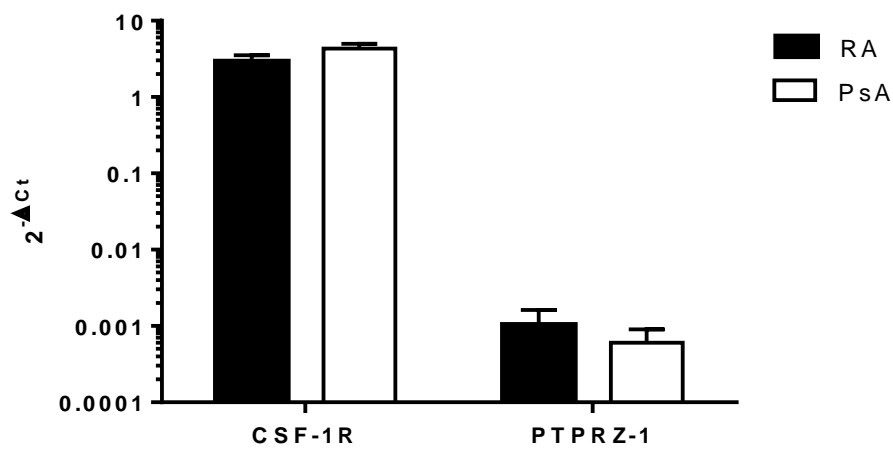

Supplement: Additional file 2: Supplementary Figures. — Figure S1. huAB1 and muAB5 antibodies block ligand-induced CSFR1 responses. a CSFR1 phosphorylation assay in IL-34 and CSF-1 stimulated CHO-CSF1R cells in the presence of increasing concentrations of huAB1. b Monocyte proliferation in IL-34- and CSF-1-stimulated NFS60 cells in the presence of increasing concentrations of muAB5. Figure S2. Circulating Ly6Clo monocyte numbers are reduced following treatment with muAB5. Graphs show the absolute number of a LyC6lo and b Ly6Chi monocytes present in blood samples obtained from muAB5- or saline-treated mice. Symbols represent values obtained from individual animals, bars represent the mean and error bars indicate the SEM. *P value <0.05; Mann-Whitney U test. Figure S3. IL-34 and CSF-1 macrophages have similar viability. Cell viability assay of monocytes from buffy coat differentiated in medium, CSF-1 or IL-34 for 1, 3 and 7 days. Data are presented as arbitrary units and represent the mean ± SEM of four independent experiments. *P < 0.05 compared to medium macrophages. Figure S4. PTP-ζ mRNA expression in synovial tissue from patients with rheumatoid arthritis (RA) or psoriatic arthritis (PsA) is almost residual compared to CSF-1R. Quantification of relative PTP-ζ and CSF1R mRNA expression in synovial tissue from 6 patients with RA and 6 patients with PsA. qPCR data are shown as 2 -ΔCt, as described in “Methods”. Data are presented as scatter plots, where each plot represents an individual value, bars represent the mean and error bars indicate the SEM. (PDF 146 kb) [file 13075_2016_973_MOESM2_ESM.pdf]
