# Supplementary material for: Colony-stimulating factor (CSF) 1 receptor blockade reduces inflammation in human and murine models of rheumatoid arthritis
Source: Arthritis Res Ther. 2016 Mar 31;18:75. doi: 10.1186/s13075-016-0973-6 (PMC4818474; doi:10.1186/s13075-016-0973-6)
Supplement: Additional file 3: — Supplementary methods. (DOCX 16 kb) [file 13075_2016_973_MOESM3_ESM.docx]

**Additional file 3: Supplementary Methods**

***Human CSF1R signal inhibition assays***

1.2x10^6^ CHO cells transfected with full-length human CSF1R (CHO-CSF1R) were incubated in U-bottom 96-well plates with increasing concentrations of huAB1 in RPMI1640/0.1% BSA for 1h on ice. Antibody coated cells were stimulated with 27 nM recombinant human CSF-1 (R&D Systems, Minneapolis, MN) or with 19.9 nM recombinant human IL-34 (provided by Five Prime Therapeutics) for 10 min. at 4C., and then lysed in Cell Lysis Buffer (Cell Signaling Technology, Danvers, MA) containing Protease and Phosphatase Inhibitors (Roche Diagnostics Corp, Indianapolis, IN). Concentrations of CSF-1 and IL-34 were chosen to correspond to the EC90 of these ligands for inducing CSF1R phosphorylation in CHO-CSF1R cells. Phosphorylated CSF1R in cell lysates was quantified using a human phospho-M-CSF R ELISA kit (R&D Systems, Minneapolis, MN). Briefly, total CSF1R in whole cell lysates of stimulated cells was captured for 2h at RT onto ELISA plates coated with 4 ug/ml anti-CSF1R and blocked with PBS/1% BSA. After washing away unbound lysate, plates were incubated with a 1/1400x dilution of anti-phosphotyrosine-HRP detection antibody for an additional 2h at RT. Plates were washed, and phosphorylated CSF1R was visualised using SuperSignal ELISA Pico Chemiluminescent Substrate (Thermo Scientific, Rockford, IL) read on an EnVision Multilabel Reader (Perkin Elmer, Waltham MA).

***mNFS60 cell proliferation assay***

mNFS60 cells were plated into half-area 96-well plates at 5x10^3^ cells/well, and cells were stimulated with 10 ng/ml of recombinant mouse CSF-1 (R&D Systems, Minneapolis, MN) or with 100 ng/ml of recombinant mouse IL-34 (R&D Systems, Minneapolis, MN) in the presence of increasing amounts of muAB5. Concentrations of mouse CSF-1 and mouse IL-34 were chosen to correspond to the the EC90 of these ligands for inducing mNFS60 cell proliferation. After a 48h incubation at 37C, ATP content of the cultures was measured with CellTiterGlo reagent (Promega, Madison, WI) according to manufacturer’s instructions. ATP content of cultures is a directly proportional to cell number, and is thus a reflection of cell proliferation/survival.

***CIA histopathology and immunohistochemistry***

At termination, hind paws, fore paws, and knees were removed and fixed in 10% neutral buffered formalin followed by decalcification in 5% formic acid. Tissues were paraffin embedded, sectioned at 8 µm and stained with toluidine blue. Hind paws, fore paws, and knees were section in the frontal plane. Six joints from each animal were processed for histopathology evaluation. The joints were scored for inflammation, pannus formation, cartilage damage, and bone resorption as previously described (Bendele et al., Arthritis & Rheumatism, 43:2648). For assessment of F4/80 stained tissues, F4/80-positive cells were counted in the entire peri-articular soft tissue and dermis of each paw or knee section. F4/80-positive cells in the bone marrow were not counted. These total counts were then normalized to counts per five 200x fields.
